# A pro-inflammatory phenotype is associated with behavioural traits in children with Prader–Willi syndrome

**DOI:** 10.1007/s00787-020-01568-7

**Published:** 2020-06-03

**Authors:** Maja Krefft, Dorota Frydecka, Gil Zalsman, Małgorzata Krzystek-Korpacka, Robert Śmigiel, Katarzyna Gębura, Katarzyna Bogunia-Kubik, Błażej Misiak

**Affiliations:** 1 Department of Psychiatry, Wroclaw Medical University, 10 Pasteur Street, 50-367 Wroclaw, Poland; 2 Child and Adolescent Division, Geha Mental Health Center, Petah Tikva, Israel; 3 Sackler Faculty of Medicine, Tel Aviv University, Tel Aviv, Israel; 4 Division of Molecular Imaging and Neuropathology, Department of Psychiatry, Columbia University, New York, USA; 5 Department of Medical Biochemistry, Wroclaw Medical University, 10 Chalubinski Street, 50-368 Wroclaw, Poland; 6 Division of Paediatrics and Rare Disorders, Department of Peadiatrics, Wroclaw Medical University, 5 Bartla Street, 50-618 Wroclaw, Poland; 7 Laboratory of Clinical Immunogenetics and Pharmacogenetics, Department of Clinical Immunology, Institute of Immunology and Experimental Therapy, Polish Academy of Sciences, 12 Rudolf Weigl Street, 53-114 Wroclaw, Poland; 8 Department of Genetics, Wroclaw Medical University, 1 Marcinkowski Street, 50-368 Wroclaw, Poland

**Keywords:** Rare disease, Inflammation, Immunity, Psychosis, Depression, Autism

## Abstract

Several lines of evidence indicate that immune-inflammatory alterations are widely observed in various mental disorders. Genetic syndromes with high risk of psychiatric disorders may constitute a model for studies investigating this phenomenon. One of such genetically determined neurodevelopmental disorders is the Prader–Willi syndrome (PWS). Therefore, we aimed to profile a broad panel of immune-inflammatory markers in patients with PWS, taking into account co-morbid psychopathology. Participants were 20 children with PWS, and 20 healthy children matched for age, sex and body mass index. Behavioural symptoms and co-occurring psychopathological symptoms were assessed using the Child Behaviour Checklist (CBCL). We found significantly elevated levels of interleukin (IL)-1β and IL-13 in patients with PWS. There were significant positive correlations between the levels of IL-1β and scores of the following externalizing and internalizing CBCL domains: withdrawn/depressed, social problems, thought problems, attention problems, delinquent and aggressive behaviour in PWS children. Moreover, higher levels of IL-13 were associated with more severe psychopathology in terms of social and attention problems as well as delinquent and aggressive behaviour. Our findings imply that subclinical inflammation, observed as elevated IL-1β and IL-13 levels, appears only in PWS patients and is correlated to several psychopathological symptoms.

## Introduction

Prader–Willi syndrome (PWS) is caused by the loss of genetic material of paternal origin from the region 15q11–q13. The loss of genetic material can occur in three mechanisms: (1) as a result of deletion of the short arm of chromosome 15 derived from father (75–80% of cases); (2) as a result of maternal uniparental disomy (mUPD—20–25% of cases) involving the presence of two homologous chromosomes coming from the mother of a person with PWS or (3) the translocation of chromosomes involving chromosome 15 and the error in the imprinting of chromosome 15 (about 5% of cases) [1].

Clinical manifestations of the syndrome include weak foetal movements in the prenatal period, decreased muscle tone in the neonatal period with subsequent disturbances in weight gain, dysmorphic features, delayed milestones and the onset of extremely uncontrollable hunger at about 2–4 years of age. Later symptoms include short statue, hypothalamic dysfunctions, hypogonadism and sleep disorders [1–3]. PWS is associated with a number of co-morbid mental disorders that include anxiety disorders, obsessive compulsive disorders (OCD) with skin picking, mood abnormalities, and psychotic disorders. Moreover, high prevalence rates of oppositional-defiant (ODD) and conduct disorder (CD), outbursts of anger and attention-deficit hyperactivity disorder (ADHD) as well as non-harmonious development of cognitive functions have been widely reported in patients with PWS. It should be noted that despite the results of intellectual functioning within the normal or borderline range in some patients with PWS, their functioning in everyday life would indicate a much lower level of intelligence [4–7]⁠.

Mechanisms underlying the co-occurrence of psychiatric conditions and activation of innate immune system in PWS patients need additional studies. It is assumed that a lack of paternally expressed genes from the 15q11–q13 region may play the role in the neurodevelopment of patients, particularly in the secretion of the cytokines involved in cell migration and proliferation as well as inflammatory processes [8]⁠. Inflammatory markers alter the metabolism and functions of neurotransmitters, neuroendocrine activity and neural plasticity. Altered regulation of this processes may lead to prolonged exposition to their action, disrupting the subtle balance in the human body, resulting in the occurrence of neuropsychiatric symptoms, such as depression, bipolar disorder (BPD) or schizophrenia [9].

Studies conducted in the population of patients with this syndrome showed elevated levels of inflammatory markers in the blood, independent of obesity [8]⁠. Subclinical inflammation has been widely associated with a number of mental disorders, including psychotic and mood disorders as well as autism spectrum disorders (ASD) that are highly prevalent in patients with PWS [8, 10–13]. Moreover, there are studies showing that several immune-inflammatory markers, such as cytokines, acute phase proteins, and specific and non-specific autoantibodies appear to be elevated in the peripheral blood of patients with these mental disorders [14, 15]. There is also a growing body of evidence that subclinical inflammation, which is not present in all patients with severe mental disorders, might hold a number of clinical implications in terms of predicting clinical and functional outcomes [16–20]. The study by Holtmann et al. on children and adolescents presenting severe affective and behavioural dysregulation also revealed alterations of the inflammatory system connecting behavioural phenotype with a pro-inflammatory state [21]. In addition, aberrant immune-inflammatory processes in mental disorders are believed to serve as potential treatment targets [22, 23]. First results of clinical trials on the modulation of inflammatory response in treatment-resistant bipolar depression show some promising outcomes with acceleration of therapeutic efficacy [24]. Treatment of major depression and schizophrenia might also benefit from the use of immunomodulatory add-on treatments [25].

The occurrence of subclinical inflammation in a number of mental disorders suggests that aberrant immune-inflammatory processes are a non-specific phenomenon that crosses traditional diagnostic boundaries [26]. However, some differences in the activation of specific immune-inflammatory processes can be observed [27]. As mentioned above, a diagnosis of PWS is a risk factor of various mental disorders, and has been associated with the activation of immune-inflammatory processes. Therefore, PWS might provide grounds to further explore which dimensions of psychopathology are related to specific aspects of subclinical inflammation. Apart from better understanding of biological mechanisms related to PWS co-morbid mental disorders, addressing this point would indicate potential targets for novel pharmacological interventions that may alleviate psychiatric symptomatology by re-establishing immunological balance. To the best of our knowledge, previous studies have not investigated the association between subclinical inflammation and behavioural traits as well as psychopathological symptoms in patients with PWS. Therefore, in this study, we aimed to examine a broad panel of inflammatory markers in patients with PWS, taking into account psychopathological manifestation.

## Methods

### Subjects

We recruited 20 PWS outpatients (11 girls and 9 boys, aged 11.5 ± 4.1 years) together with a group of 20 typically developing children (11 boys and 9 girls, aged 10.25 ± 2.9 years) as healthy controls (HC). Every PWS patient was under the care of a dietician and the vast majority of them were not overweight. All PWS patients were on growth hormone (GH) replacement therapy. The group of PWS patients was recruited through advertisements sent by the Polish PWS Association of Parents and the Foundation for Children with Rare Diseases. Additionally, a register of Department of Genetics (Wroclaw Medical University, Wroclaw, Poland) was used to send invitations to participate in the study. Parents and caregivers of children with a diagnosis of PWS from all over the country were invited by means of a letter/e-mail messages/telephone calls or lectures during family meetings for relatives of PWS patients, describing the purpose of the study. Recruitment was not conducted in psychiatric facilities. Due to the low prevalence of the PWS, we could not avoid a certain bias in the recruitment. All patients with PWS diagnosis with norm or borderline intellectual functioning willing to participate were included in the study. Intellectual functioning of children was assessed by the Wechsler Intelligence Scale for Children-revised (WISC-R) [28] and Wechsler Adult Intelligence Scale-revised (WAIS-R) [29].The patients attended public or integrating schools. HCs had no past or current psychiatric illness. They were recruited from local social groups for parents and during the seminar about research and rare diseases that took place at a public school in Wroclaw, Poland.

The study received approval of the Ethics Committee at Wroclaw Medical University. Informed, written consent was collected from parents and legal guardians of children as well as from patients aged over 16 years.

### Measures

Participants were referred for physical and neuropsychiatric examination that included: (1) physical examination collecting basic health parameters; (2) an author’s questionnaire of socio-demographic characteristics and basic information consisting of semi-open questions about early development and medical history, education career as well as the questionnaire about eating habits and disorders with the criteria for a clinical diagnosis of PWS; (3) the Child Behaviour Checklist for school-age children (CBCL/6–18) and (4) the Polish version of the Mini-International Neuropsychiatric Interview for Children and Adolescents (MINI Kid) supplementary questionnaire.

#### Physical examination

Basic health parameters, such as systolic and diastolic blood pressure (SBP and DBP), were measured using an electronic sphygmomanometer with inflatable cuff adapted for measurements in children. Waist and hip circumference were recorded for each participant. Waist circumference was taken starting at the top of the patient’s hip bone, then bringing the measuring tape around the body leveled with belly button. Hip circumference was the distance around the largest part of the patient’s hips. Body mass and height were measured in light clothing and without shoes to calculate the body mass index (BMI).

#### The CBCL/6–18

The Polish translation of the CBCL was completed by parents and caregivers to obtain the syndrome scale scores on children’s competences as well as internalizing and externalizing problems [30]. The CBCL is a widely used tool for assessing the various aspects of children's behaviour in the observation of parents/guardians. It consists of empirically based syndrome scales: anxious/depressed; withdrawn/depressed; somatic complaints, social problems, thought problems, attention problems, rule breaking behaviour, aggressive behaviour as well as six DSM-oriented scales: depressive problems, anxiety problems, somatic problems, attention deficit/hyperactivity problems, oppositional defiant problems and conduct problems. Scoring for the CBCL is based on statistical groupings of sets of behaviours that commonly appear collectively [31].

#### The MINI Kid

The Polish version of the MINI Kid was completed by children and parents as screening for most common and clinically relevant disorders or disorder subtypes in paediatrics mental health. The MINI Kid is a structured diagnostic interview for DSM-IV and ICD-10 psychiatric disorders in children and adolescents. Given impaired cognitive capacity of some patients with PWS, the interview was taken in the presence and supervision of parents and carers of all participants. The MINI Kid assesses the 30 most prevalent and clinically significant disorders or disorder subtypes in children and adolescents [32]⁠. Assessment was performed by one trained clinician (M. K.), who was a child and adolescent psychiatry trainee (4th and 5th year of training).

#### Immuno-inflammatory markers

The levels of immuno-inflammatory markers were measured in the fasting serum using the Multiplex Bioplex immunoassays that enable quantitative assessment of multiple biomarkers. The Luminex^®^ 200™ platform (Luminexcorp, USA) was used, and validated 27-plexes for simultaneous measurement of the following markers: interleukin (IL)-1RA, IL-1β, IL-2, IL-4, IL-5, IL-6, IL-7, IL-8, IL-9, IL-10, IL-12, IL-13, IL-15, IL-17, interferon-γ (IFN-γ), eotaxin, IFN-γ-induced protein 10 (IP-10), fibroblast growth factor-2 (FGF-2), granulocyte colony-stimulating factor (G-CSF), granulocyte–macrophage colony-stimulating factor (GM-CSF), monocyte chemoattractant protein-1 (MCP-1), macrophage inflammatory protein-1α (MIP-1α), macrophage inflammatory protein-1α (MIP-1β), tumour necrosis factor α (TNF-α), vascular endothelial growth factor A (VEGF-A), platelet-derived growth factor with two B subunits (PDGF-BB) and Regulated on Activation, Normal T-cell Expressed and Secreted (RANTES) according to manufacturer's instructions (BioRad, USA). Immune-inflammatory markers that had concentrations under detection levels in more than half of the participants from the group of PWS patients or controls were excluded from data analysis (IL-12, IL-15, RANTES, GM-CSF and IFN-γ). Intra-assay coefficients of variations (CVs) for all studied cytokines/growth factors were ≤ 10% while inter-assay CVs were ≤ 16%.

### Statistics

Between-group differences in the distribution of categorical variables were examined using the *χ*^2^ test. In case of continuous variables, the Kolmogorov–Smirnov test was used to assess normality of data distribution. *T* tests were used for the comparison of continuous variables with normal distribution. Otherwise, the Mann–Whitney *U* test was used. Correlations were assessed using the Spearman rank correlation coefficients. Due to several bivariate comparisons (194 bivariate comparisons and correlations), the Benjamini–Hochberg correction for multiple testing was applied to the level of significance (the false discovery rate was set at 25%). A total of 194 *p* values were ranked from smallest to largest. Subsequently, the Benjamin–Hochberg critical value was calculated according to the formula: (*i*/*m*)*Q*, where *i* is the rank, *m* is the total number of tests and *Q* is the false discovery rate of 25% (0.25). All *p* values below the Benjamini–Hochberg critical value of 0.005 were considered significant in bivariate tests (*p* ≤ 0.003). Between-group differences in the levels of inflammatory markers were further tested using the analysis of co-variance (ANCOVA). Age, sex and waist circumference were included as co-variates. The alpha criterion level was set at 0.05 in the ANCOVA. The Statistical Package for Social Sciences, version 20 (SPSS Inc., Chicago, IL, USA) was used to perform all analyses.

## Results

The general characteristics of the sample are shown in Table 1. There was no statistically significant difference between both groups of participants in age, the number of males and females as well as anthropometric parameters. There were significantly higher levels of IL-1β (0.4 ± 0.2 vs. 0.2 ± 0.1 pg/ml, *p* < 0.001) and IL-13 (2.9 ± 1.4 vs. 1.5 ± 0.6 pg/ml, *p* < 0.001) in patients with PWS compared to HCs (Table 2). The levels of IL-1RA (275.3 ± 248.1 vs. 65.8 ± 66.5 pg/ml, *p* = 0.017), IL-1β (0.5 ± 0.1 vs. 0.2 ± 0.01 pg/ml, *p* = 0.009) and IL-6 (1.0 ± 0.3 vs. 0.3 ± 0.2 pg/ml, *p* = 0.011) were also significantly higher in two patients with BMI over the 90th percentile (1 male and 1 female) compared to HCs. The levels of other immune-inflammatory markers were similar in both groups of participants. The ANCOVA revealed that the difference in IL-1β levels remained significant (*F* = 21.40, *p* < 0.001) after co-varying for age (*F* = 6.53, *p* = 0.015), sex (*F* = 1.37, *p* = 0.250) and waist circumference (*F* = 7.09, *p* = 0.012). Similarly, the difference in IL-13 levels was significant (*F* = 16.90, *p* < 0.001) after adjustment for age (*F* = 0.81, *p* = 0.376), sex (*F* = 0.167, *p* = 0.685) and waist circumference (*F* = 0.31, *p* = 0.579).

**Table 1:** General characteristics of participants

	PWS (*n* = 20)	HCs (*n* = 20)	*p*
Age (years)	11.5 ± 4.1	10.25 ± 2.9	0.461
Sex, M/F (%)	9 (45.0)/11 (55.0)	11 (55.0)/9 (45.0)	0.527
BMI percentile	48.8 ± 30.8	48.3 ± 37.1	0.967
Waist circumference (cm)	67.1 ± 14.8	64.4 ± 15.3	0.309
Hip circumference (cm)	78.8 ± 17.1	75.45 ± 14.8	0.607
Mood disorders, *n* (%)	5 (20.0)	–	–
Anxiety disorders, *n* (%)	7 (35.0)	–	–
OCD, *n* (%)	9 (45.0)	–	–
Tics syndrome, *n* (%)	1 (5.0)	–	–
ADHD, *n* (%)	6 (30.0)	–	–
Conduct disorder, *n* (%)	1 (5.0)	–	–
ODD, *n* (%)	8 (40.0)	–	–
Psychotic disorder, *n* (%)	1 (5.0)	–	–
Adjustment disorder, *n* (%)	2 (10.0)	–	–
ASD, *n* (%)	6 (30.0)	–	–
Co-morbidities according to MINI-Kid		–	–
0, *n* (%)	3 (15.0)	–	–
1, *n* (%)	4 (20.0)	–	–
2–3, *n* (%)	9 (45.0)	–	–
≥ 4, *n* (%)	4 (20.0)	–	–

The ICD-10 diagnoses established based on the Mini KID interview were presented in table

*M* male, *F* female, *BMI* body mass index, *HCs* healthy controls, *PWS* Prader–Willi syndrome patients, *ADHD* attention deficit hyperactivity disorder, *OCD* obsessive–compulsive disorder, *ODD* oppositional-defiant disorder, *ASD* autism spectrum disorder

**Table 2:** The levels of inflammatory markers in PWS patients and healthy controls

	*n* (PWS/HCs)	PWS (*n* = 20)	HCs (*n* = 20)	*p*
IL-1β	(20/20)	0.4 ± 0.2	0.2 ± 0.1	**< 0.001**
IL-1RA	(20/20)	106.6 ± 99.3	65.8 ± 66.5	0.010
IL-2	(20/20)	2.5 ± 0.6	2.2 ± 0.8	0.213
IL-4	(20/20)	3.9 ± 1.1	3.3 ± 1.1	0.100
IL-5	(20/19)	6.9 ± 4.2	7.4 ± 4.3	0.687
IL-6	(19/18)	0.4 ± 0.3	0.3 ± 0.2	0.086
IL-7	(20/20)	12.7 ± 4.2	12.7 ± 4.2	0.988
IL-8	(20/20)	6.9 ± 3.6	6.8 ± 5.2	0.495
IL-9	(20/20)	164.3 ± 59.5	138.0 ± 70.1	0.314
IL-10	(18/16)	2.15 ± 1.4	2.2 ± 1.1	0.984
IL-13	(20/20)	2.9 ± 1.4	1.5 ± 0.6	**< 0.001**
IL-17	(20/20)	11.1 ± 4.3	11.5 ± 3.9	0.565
IP-10	(20/20)	346.7 ± 239.3	394.1 ± 333.3	0.779
MCP-1	(20/20)	32.4 ± 14.4	26.7 ± 13.9	0.208
MIP-1α	(20/20)	1.5 ± 0.7	2.1 ± 1.5	0.192
MIP-1β	(20/20)	46.8 ± 5.5	49.4 ± 7.2	0.383
PDGF-BB	(20/20)	3025.0 ± 1204.2	2571.8 ± 1015.7	0.206
TNF-α	(20/20)	11.9 ± 2.4	11.3 ± 2.1	0.277
Eotaxin-1	(20/20)	67.0 ± 28.0	68.6 ± 23.1	0.852
FGF-2	(20/20)	18.6 ± 3.8	18.6 ± 3.4	0.953
G-CSF	(20/20)	158.0 ± 41.2	170.2 ± 53.1	0.422

Data expressed as mean ± SD. The levels of inflammatory markers were represented in pg/ml

Significant differences after the Benjamini–Hochberg correction were marked with bold characters (*p* ≤ 0.003)

*IL* interleukin, *IP-10* interferon-γ induced protein 10, *MCP-1* monocyte chemoattractant protein-1, *MIP-1α* macrophage inflammatory protein-1α, *MIP-1 β* macrophage inflammatory protein-1β, *TNF-α* tumour necrosis factor α, *PDGF-BB* platelet-derived growth factor with two B subunits, *FGF-2* fibroblast growth factor-2, *G-CSF* granulocyte colony-stimulating factor

Analysis of bivariate correlations (Table 3) revealed significant positive correlations between the levels of IL-1β and scores of the following CBCL domains: withdrawn/depressed (*r* = 0.515, *p* = 0.001), social problems (*r* = 0.623, *p* < 0.001), thought problems (*r* = 0.530, *p* < 0.001), attention problems (*r* = 0.650, *p* < 0.001), delinquent (*r* = 0.588, *p* < 0.001) and aggressive behaviour (*r* = 0.518, *p* = 0.001) in PWS children. Moreover, higher levels of IL-13 were associated with more severe psychopathology in terms of social (*r* = 0.490, *p* = 0.001) and attention (*r* = 0.466, *p* = 0.002) problems as well as delinquent (*r* = 0.483, *p* = 0.002) and aggressive behaviour (*r* = 0.456, *p* = 0.003). Correlations between the levels of other immune/inflammatory markers and the levels of various psychopathological symptoms did not reach statistical significance.

**Table 3:** Bivariate correlations between the levels of inflammatory markers and the measures of psychopathology in PWS patients

	Anxious/depressed	Withdrawn/depressed	Somatic complaints	Social problems	Thought problems	Attention problems	Delinquent behaviour	Aggressive behaviour
IL-1β	0.225/0.163	**0.515/0.001**	0.386/0.014	**0.623/< 0.001**	**0.530/< 0.001**	**0.650/< 0.001**	**0.588/< 0.001**	**0.518/0.001**
IL-1RA	0.160/0.324	0.130/0.422	0.122/0.455	0.409/0.009	0.308/0.053	0.225/0.164	0.405/0.009	0.321/0.043
IL-2	0.053/0.745	0.179/0.268	0.0.22/0.894	0.233/0.148	0.227/0.160	0.284/0.076	0.348/0.028	0.221/0.171
IL-4	0.192/0.236	0.348/0.028	0.304/0.056	0.220/0.173	0.192/0.236	0.348/0.028	0.304/0.056	0.269/0.093
IL-5	− 0.276/0.089	− 0.200/0.223	− 0.202/0.217	− 0.090/0.587	− 0.191/0.243	0.039/0.814	− 0.004/0.982	0.015/0.930
IL-6	− 0.094/0.579	0.028/0.867	− 0.051/0.765	0.266/0.111	0.274/0.100	0.311/0.061	0.338/0.041	0.292/0.079
IL− 7	− 0.108/0.509	− 0.119/0.463	− 0.171/0.293	− 0.004/0.981	− 0.043/0.793	0.104/0.525	0.047/0.773	0.007/0.965
IL-8	− 0.123/0.473	− 0.021/0.899	− 0.111/0.495	0.149/0.360	− 0.131/0.420	0.091/0.575	0.046/0.779	0.082/0.161
IL-9	0.130/0.424	0.312/0.050	0.219/0.175	0.203/0.209	0.156/0.338	0.115/0.478	0.232/0.150	0.253/0.116
IL-10	− 0.067/0.702	0.018/0.918	− 0.111/0.527	0.051/0.770	− 0.010/0.954	0.038/0.827	0.216/0.213	0.150/0.388
IL-13	0.117/0.471	0.356/0.024	0.174/0.284	**0.490/0.001**	0.387/0.014	**0.466/0.002**	**0.483/0.002**	**0.456/0.003**
IL-17	− 0.111/0.494	− 0.129/0.426	− 0.187/0.248	− 0.042/0.798	− 0.100/0.540	0.041/0.800	0.028/0.862	− 0.048/0.771
IP-10	− 0.083/0.612	− 0.342/0.031	− 0.068/0.678	− 0.045/0.782	− 0.044/0.788	− 0.106/0.518	− 0.094/0.564	0.002/0.993
MCP-1	− 0.020/0.904	0.084/0.604	− 0.102/0.530	0.147/0.366	0.166/0.477	0.123/0.448	0.151/0.352	0.127/0.436
MIP-1α	− 0.141/0.412	− 0.302/0.059	− 0.250/0.119	− 0.074/0.650	− 0.152/0.351	− 0.160/0.323	− 0.133/0.413	− 0.079/0.629
MIP-1β	− 0.232/0.150	− 0.154/0.342	− 0.212/0.189	− 0.113/0.489	− 0.112/0.490	− 0.106/0.515	0.062/0.704	0.008/0.963
PDGF-BB	0.092/0.595	0.075/0.665	0.026/0.874	0.102/0.529	0.073/0.657	0.019/0.909	0.138/0.397	0.030/0.855
TNF-α	− 0.012/0.939	0.112/0.491	0.094/0.563	0.145/0.373	0.261/0.104	0.223/0.166	0.382/0.015	0.188/0.244
Eotaxin-1	− 0.284/0.075	− 0.132/0.416	− 0.280/0.080	− 0.108/0.507	− 0.123/0.449	0.021/0.897	− 0.023/0.899	− 0.050/0.761
FGF-2	− 0.063/0.700	− 0.081/0.618	− 0.118/0.468	− 0.013/0.937	− 0.054/0.739	0.046/0.776	0.017/0.916	− 0.018/0.911
G-CSF	− 0.167/0.304	− 0.187/0.247	− 0.214/0.185	− 0.011/0.946	− 0.063/0.701	0.093/0.568	0.043/0.793	0.046/0.779

Data expressed as *r*/*p* value

Significant correlations after the Benjamini–Hochberg correction were marked with bold characters

(*p* ≤ 0.003)

*IL* interleukin, *IP-10* interferon-γ induced protein 10, *MCP-1* monocyte chemoattractant protein-1, *MIP-1α* macrophage inflammatory protein-1α, *MIP-1 β* macrophage inflammatory protein-1β, *TNF-α* tumour necrosis factor α, *PDGF-BB* platelet-derived growth factor with two B subunits, *FGF-2* fibroblast growth factor-2, *G-CSF* granulocyte colony-stimulating factor

## Discussion

In this study, we found significantly higher serum levels of IL-1β and IL-13 in PWS patients compared to healthy controls, even after adjustment for potential confounding factors. Patients with PWS and BMI over 90th percentile had also elevated levels of IL-1β, IL-1RA and IL-6. Both groups were matched for age, sex and anthropometric parameters. In this regard, our findings suggest that a pro-inflammatory state in PWS patients cannot be attributed to overweight or obesity. Until recently, the levels of inflammatory cytokines in PWS have been described in the context of obesity or metabolic syndrome. Caixàs et al. [33] were the first to show that adult patients with PWS have higher levels of inflammatory markers when compared to healthy controls. Higher plasma concentrations of C-reactive protein (CRP), IL-18, and IL-6 were described in this study. Moreover, high IL-18 levels were related to low testosterone concentrations in PWS males [33]. Viardot et al. [34] reported increased activation of the innate immune system with significantly higher IL-6 and non-significantly higher CRP in patients with PWS and obesity compared to adiposity-matched subjects. These results were also associated with obstructive sleep apnoea syndrome and appeared to be a contributor to reduced life expectancy due to cardiovascular disease in PWS [34]. Another study showed elevated blood levels of four chemokines: MCP1, macrophage-derived chemokine (MDC), eotaxin and RANTES in PWS patients compared to their unaffected siblings, independent of obesity [8].

Evidence suggests the existence of cytokine alterations in several mental disorders. IL-1β, a pro-inflammatory cytokine is described in the context of psychiatric conditions, such as depression [35, 36]⁠ and schizophrenia, where it may predict a severity of general and global symptomatology [37] or ASD [13] the features of which are especially present in PWS patients. A recent meta-analysis of pro-inflammatory cytokines provided evidence for elevated levels of IL-1β, IL-6, TNF-α and IFN-γ in patients with ASD in comparison with healthy subjects [13]. Interestingly, higher blood levels of IL-1β may be a potential biological marker of increased risk of developing mild to moderate ASD [13]. Studies on ASD patients suggest that immune activation may be linked to more impaired behavioural symptomatology, such as social withdrawal [38]⁠. IL-1β has also been characterized as an early marker of later psychological problems and psychomotor disabilities in children from the study by Voltas et al. on healthy newborns [39]⁠. Both ASD and schizophrenia share some cellular mechanisms involved in the regulation of gene expression and synaptic plasticity, with a critical role of inflammation. Similarities in both conditions include high heritability, developmental delay (even before the onset of psychosis in schizophrenia patients), impaired pattern of behaviour and activities as well as difficulties in interpersonal relationships. The presence of elevated inflammatory factors, in particular IL-1β, may indicate a common aetiology of these disorders [40]⁠. Another meta-analysis by Goldsmith et al. (2016) again pointed the levels of IL-1β and soluble IL-2 receptor (sIL-2R) to be significantly elevated in schizophrenia and BPD in chronically ill patients [27]⁠. However, a recent meta-analysis of eight studies investigating pro-inflammatory cytokines in children and adolescents with depressive disorders revealed only a trend toward significantly higher levels of peripheral TNF-α [10, 41]⁠. Nevertheless, animal model studies conducted on intracerebroventricular IL-1β injection indicate the role of IL-1β in developing depressive-like behaviours by disrupting the immunological homeostasis [42]. IL-1β is a strong modulator of corticotrophin-releasing hormone causing hypothalamic–pituitary–adrenal axis activation with increased secretion of adrenocorticotropic hormone and corticosterone, which both occur in major depression. Another effect of IL-1β is related to stimulation of fibroblasts to produce prostaglandin E2 which is described as the mediator of inflammatory processes [42].

Moreover, it has been reported that the IL-1β rs16944 polymorphism is associated with neurostructural alterations accompanied by neurocognitive dysfunctions in youth with BPD [43]. Also, the IL-1β rs16944 polymorphism has been associated with reduced hippocampal volume, white matter and bilateral frontotemporal grey matter deficits as well as and ventriculomegaly in patients with schizophrenia [44]⁠. Further research on PWS patients could include younger patients to determine whether IL-1β is already elevated during infancy and correlates with poorer neurocognitive functioning and more psychopathological symptoms in adolescence and adulthood [39]⁠.

Another finding of our study is related to elevated level of anti-inflammatory cytokine IL-13 that among other Th-2 cytokines enhances the humoral response by activating cells to express antibodies. It might be hypothesized that elevated levels of IL-13 appear as a response to subclinical inflammation. Previous meta-analyses addressing the levels of cytokines in patients with mental disorders also demonstrated that the levels of both pro- and anti-inflammatory cytokines appear to be elevated [45, 46]. In a rodent model, IL-13 had negative impact on neuron viability in some regions of the brain and as it was able to induce neuroinflammation of the central nervous system by destruction of microglia cells, which would stand for the development of both neurodegenerative diseases and psychiatric disorders [47]⁠.

The mechanisms underlying the observation that TNF-α and IL-6 were not elevated in our sample of patients with PWS in contrast to other studies on this population, as well as on patients with schizophrenia, bipolar mania or major depressive disorder (MDD), may be due to the fact that the vast majority of our patients were not overweight (only two patients with BMI over 90^th^ percentile for sex and age). Studies showing elevated levels of TNF-α or IL-6 in PWS patients, mainly included obese patients [48–50]. In addition, studies on children with PWS receiving GH have shown the effect of GH supplementation on the reduction of cytokine levels [51, 52]. In contrast, the study conducted by Viardot et al. showed significantly elevated IL-6 levels in PWS patients, but none of them was receiving GH [34]. Further studies are required to confirm whether maintenance of normal body weight together with GH supplementation results in lower TNF-α and IL-6 levels in PWS paediatric patients.

To the best of our knowledge, this is the first study investigating correlations between the activation of innate immune system and psychopathological symptoms in PWS patients. We found several strong correlations of which two are interesting in the context of psychotic disorders and autistic features. Higher levels of IL-1β were associated with more severe symptoms of withdrawal/depression and thought problems. These scales were described as predictive for the development of psychotic disorders, ASD and BPD [53–55]⁠. Other correlations with IL-1β included social and attention problems as well as aggressive and delinquent behaviours. Strong and moderate correlations for IL-13 were found in social and attention problems as well as in delinquent and aggressive behaviours.

Our findings are consistent with the results of previous studies on the prevalence of mental disorders in the population of PWS patients [56]. In groups of patients with higher intellectual functioning, increased frequency of OCD symptoms, anxious-depressed and attention disorders as well as thought problems were described more often than in lower IQ children with PWS. Also social problems, aggression and anxiety were more commonly reported in the higher IQ PWS group [57]. Second most common condition present in our study group was ODD with very characteristic temper tantrums, aggressive impulsiveness and anger outbursts [58]. Insistence on routine with repetitive behaviours together with difficulties in social interaction, similar to those in ASD reported in other studies were also present in every third of our PWS patients [59].

This study has some limitations. Firstly, our sample was limited to 20 patients and thus type I error cannot be excluded. An important limitation of our study is that we used BMI as a measure of adiposity. It should be noted that the BMI percentile often remains within the normal range for age and sex and may not correspond with heightened percentage of body fat in PWS patients. It has been found that patients with PWS have unusual fatness pattern with reduced lean tissue and increased adiposity [60]. All of the PWS patients in our sample were also on GH replacement therapy which could affect the body composition and fat percentage as well as patients’ height, influencing BMI. At this point, it is also important to note that we were unable to determine whether subclinical inflammation appears only in a certain subgroup of patients with PWS. Secondly, the vast majority of patients included in our study had a diagnosis of PWS due to deletion (19 out of 20 patients). Notably, the prevalence of mUPD among PWS patients has been estimated at 20–30% and this mechanism has been particularly related to a number of co-morbid psychiatric disorders [61]. Moreover, a cross-sectional study design does not allow to conclude regarding causal associations between subclinical inflammation and co-morbid psychopathological symptoms in PWS patients. Finally, it should be noted that while genetic syndromes with frequent psychiatric comorbidities may serve as a model system, the generalizability of findings might be questionable.

There are many questions regarding the use of cytokines as biomarkers in psychiatric diseases due to high overlap of cytokines levels between patients at different illness phases and healthy controls, as well as due to the multitude of environmental and genetic factors impacting cytokine levels [62, 63]. Reliable comparisons of cytokines levels require taking into account multiple confounding factors, such as: obesity, age, physical activity, medication, smoking status, concomitant disorders (e.g., allergies or autoimmune disorders). Specifically, PWS is often associated with inflammatory processes and various co-morbidities, such as cardiovascular disease, hypertension or diabetes mellitus since early childhood. This challenge can also be exemplified by the levels of IL-1β in PWS patients, which are significantly elevated in this study, but were below detection limit in others [8]. Due to different results obtained in cytokine research in PWS patients, meta-analytic studies could be helpful in resolving conflicting findings.

## Conclusion

In summary, the results of our study indicate that subclinical inflammation, in terms of elevated IL-1β and IL-13, appears in PWS patients regardless of overweight or obesity. These observations might be related to a risk of developing various psychiatric co-morbidities and are in agreement with studies exploring subclinical inflammation in various populations of patients with severe mental disorders. This agreement might also suggest that subclinical inflammation in various mental disorders might be a downstream effector of other pathophysiological processes. However, the exact mechanisms of our observations remain unknown. Longitudinal studies investigating inflammatory signatures in children at early developmental periods are needed to establish causal inferences. Moreover, the comparison of inflammatory markers in patients with PWS due to various genetic mechanisms might provide further insights into potential mechanisms of our observations.
